# Unraveling Hepcidin Plasma Protein Binding: Evidence from Peritoneal Equilibration Testing

**DOI:** 10.3390/ph12030123

**Published:** 2019-08-23

**Authors:** Laura E. Diepeveen, Coby M. Laarakkers, Hilde P.E. Peters, Antonius E. van Herwaarden, Hans Groenewoud, Joanna IntHout, Jack F. Wetzels, Rachel P.L. van Swelm, Dorine W. Swinkels

**Affiliations:** 1 Department of Laboratory Medicine, Radboud University Medical Center, 6525 Nijmegen, The Netherlands; 2 Department of Nephrology, Isala Hospital, 8025 Zwolle, The Netherlands; 3 Department of Health Evidence, Radboud University Medical Center, 6525 Nijmegen, The Netherlands; 4 Department of Nephrology, Radboud University Medical Center, 6525 Nijmegen, The Netherlands

**Keywords:** iron homeostasis, hepcidin, protein binding, peritoneal dialysis

## Abstract

Peptide hormone hepcidin regulates systemic iron metabolism and has been described to be partially bound to α2-macroglobulin and albumin in blood. However, the reported degree of hepcidin protein binding varies between <3% and ≈89%. Since protein-binding may influence hormone function and quantification, better insight into the degree of hepcidin protein binding is essential to fully understand the biological behavior of hepcidin and interpretation of its measurement in patients. Here, we used peritoneal dialysis to assess human hepcidin protein binding in a functional human setting for the first time. We measured freely circulating solutes in blood and peritoneal fluid of 14 patients with end-stage renal disease undergoing a peritoneal equilibration test to establish a curve describing the relation between molecular weight and peritoneal clearance. Calculated binding percentages of total cortisol and testosterone confirmed our model. The protein-bound fraction of hepcidin was calculated to be 40% (±23%). We, therefore, conclude that a substantial proportion of hepcidin is freely circulating. Although a large inter-individual variation in hepcidin clearance, besides patient-specific peritoneal transport characteristics, may have affected the accuracy of the determined binding percentage, we describe an important step towards unraveling human hepcidin plasma protein binding in vivo including the caveats that need further research.

## 1. Introduction

The peptide hormone hepcidin, produced by hepatocytes, regulates iron entry into the circulation and iron distribution throughout the body by degradation of the cellular iron exporter ferroportin [1]. Hormones can circulate freely or partially bound to carrier proteins in the human body [2,3]. These carrier proteins allow transport of non-soluble hormones through the blood plasma, prevent excretion, and function as a reservoir thereby regulating hormone bioavailability. The free hormone hypothesis indicates that most hormones are not capable of exerting their physiological effect in a protein-bound form [2,4,5,6]. Hence, for partially bound hormones, such as thyroxine, testosterone, triiodothyronine, and cortisol, assays were designed to specifically quantify the bioactive free hormone concentration [5,7,8,9].

It is suggested that hepcidin in blood is partially bound to both α2-macroglobulin (α2M) and albumin [10]. However, there is large controversy on the subject since the reported plasma protein-bound fraction of hepcidin varies between <3% and ≈89% [10,11,12]. Consequently, it is uncertain to what degree currently available analytical assays quantify (only) bioactive hepcidin. Differences in extent of quantification of the freely circulating hepcidin relative to the protein bound compound between assays may influence interpretation and comparability of hepcidin measurement and hampers recent achievements on standardization of hepcidin assays [13]. Characterization of the measured analyte will add the final aspect to the metrological traceability chain of hepcidin, which describes an unbroken calibration hierarchy from a measurement result to a defined reference in SI units. Additionally, unraveling the protein binding properties of hepcidin will improve our understanding of its biology and will allow its correct quantification, which is essential in the assessment and interpretation of the pharmacokinetic and pharmacodynamic effects of hepcidin agonist or antagonist therapies [1,14]. To address these issues, we aimed to study the degree of hepcidin protein binding in a functional human setting for the first time, rather than using previously reported in vitro (research) techniques [10,11], mice in vivo studies [11], or human ex vivo serum analysis [12]. Especially since current in vitro techniques to assess free hormone concentrations in diagnostic medicine, such as equilibrium dialysis and ultrafiltration methods, have limitations amongst which are risk for leakage, non-specific absorption of the hormone leading to low reproducibility, and bias of results, osmotic effects or the Gibbs-Donnan effect [15].

To circumvent the limitations of in vitro studies, we aimed to unravel hepcidin plasma protein binding with a new approach using the principle of peritoneal dialysis (PD). PD is a treatment option for patients with end-stage renal disease (ESRD), in which the peritoneal cavity is filled with dialysis fluid. During a certain dwell time, plasma components are transported into the peritoneal cavity. Transport of molecules is mainly driven by diffusion and ultrafiltration and depends on peritoneal membrane characteristics [16]. To assess these individual characteristics, all patients treated with PD frequently undergo a standardized and highly reproducible peritoneal equilibration test (PET) [17,18,19]. During a four-hour dialysis exchange, the peritoneal membrane transport function of a patient treated with PD is characterized to classify them as either a high, low, or average transporter in order to optimize the dwell time [17]. These peritoneal equilibration rates reflect peritoneal clearance (PC) and can be calculated using the dialysate-to-plasma ratio (D/P ratio) for any given solute that is transported from the peritoneal capillary blood into dialysate fluid. The transport rate over the peritoneal membrane into the peritoneal cavity is largely size-selective and thus the PC is inversely related to the radius or molecular weight (MW) of a molecule, regardless of molecular properties such as charge or hydrophobicity [20,21,22,23,24]. Therefore, the transport rate of small molecules that are bound to large plasma proteins will be determined by the size of the complex. This has been verified using the protein-bound solute p-cresol (MW: 108 Da), since its PC was found to be similar to β2-microglobulin (MW: 11,815 Da) [25]. Although a high PC was expected based on the low MW of p-cresol, its clearance was much lower, confirming protein-binding.

In this study, we measured hepcidin, along with known freely circulating and protein-bound solutes, in blood and dialysate of patients with ESRD undergoing PET. We used this model to explore its suitability to quantify hormone binding, and therefore to unravel the extent of hepcidin binding in the circulation, by studying if the measured PC of hepcidin fits the curve describing the relation between MW and PC of free circulating solutes.

## 2. Results

In total, 14 patients with ESRD participated in this study. Mean age was 67 years, total creatinine clearance was between 56.4 and 129.5 L/week/1.73 m^2^, and residual creatinine clearance was between 25.7 and 100.0 L/week/1.73 m^2^ (Table 1). The start time of the PET after an overnight dwell was between 8:30 a.m. and noon. Four patients used prednisone (Table 1), which interferes with both total and free cortisol measurement due to metabolism into prednisolone. Therefore, cortisol measurements were not performed in these patients.

**Figure 1:**
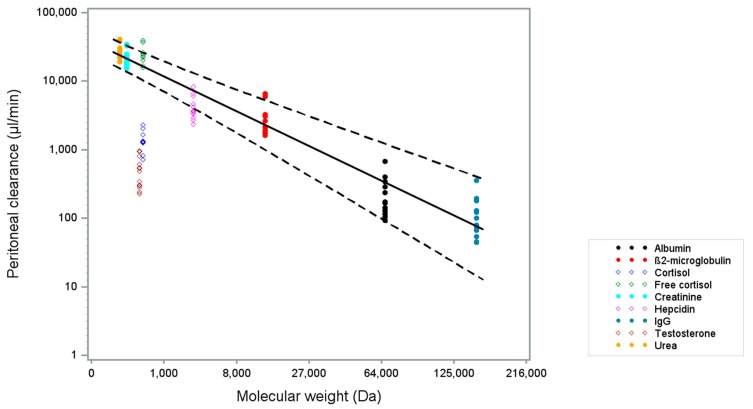
Peritoneal clearances (µL/min) as a function of molecular weight (Da). The logarithmically transformed peritoneal clearance (y-axis) of each specific analyte for each patient was plotted against the cube root of their molecular weight (x-axis). Based on the known free circulating analytes, i.e., urea, creatinine, β2-microglobulin, albumin, and IgG (solid dots), a curve was established describing the relation between clearance and molecular weight (solid line) with a 95% prediction interval (dashed lines). Hepcidin, free cortisol, total cortisol, and total testosterone measurements (open dots) were plotted in the figure thereafter.

We measured and subsequently calculated the PC of free circulating solutes urea, creatinine, β2-microglobulin, albumin, and IgG (Table 2), and plotted the logarithmically transformed PC of each analyte against its MW for each patient. By applying a linear mixed model, we obtained a curve with a 95% prediction interval describing the relation between MW and PC of free circulating solutes (Figure 1, solid dots). We found the equation defining the curve to be:

Log10 (PC) = 4.5744 – 0.05063 * ∛MW.(1)

We tested the validity of our model by measuring both total (i.e., free and protein-bound) and free cortisol and total testosterone. As expected, the PC measurements of total cortisol (Figure 1, open blue dots) and total testosterone (Figure 1, open brown dots) plotted against the MW of the unbound molecules were below the free solutes curve, confirming their predominantly bound status. Their binding percentages were calculated to be 91% (±3%) and 97% (±1.5%), respectively, corresponding to what is described in literature [6,9]. The mean of free cortisol PC measurements (Figure 1, open green dots) fell within the 95% confidence interval to the free solutes curve, confirming its unbound status.

We subsequently plotted the hepcidin PC measurements against the MW of hepcidin in the graph (Figure 1, open pink dots). Interestingly, the mean PC for hepcidin was found to lie below the established free solute curve. The calculated binding percentage is 40% (±23%), suggesting that hepcidin circulates in a more unbound than bound status, with a large inter-individual variability. Since several hepcidin PC measurements fell within the 95% confidence interval, we can exclude a predominantly protein-bound status of hepcidin, as we found for total cortisol and testosterone.

**Figure 2:**
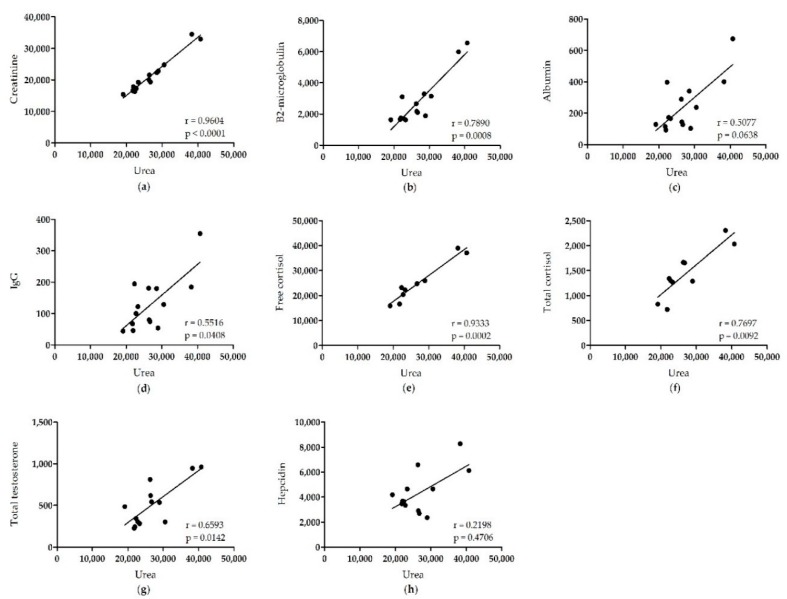
Correlations among the peritoneal clearance of urea with all analytes in patients with end-stage renal disease undergoing a peritoneal equilibration test. The peritoneal clearance of urea was correlated to the peritoneal clearance of (**a**) creatinine, (**b**) β2-microglobulin, (**c**) albumin, (**d**) IgG, (**e**) free cortisol, (**f**) total cortisol, (**g**) total testosterone, and (**h**) hepcidin. The strength of the correlation was measured using Spearman’s correlation coefficient (r).

**Figure 3:**
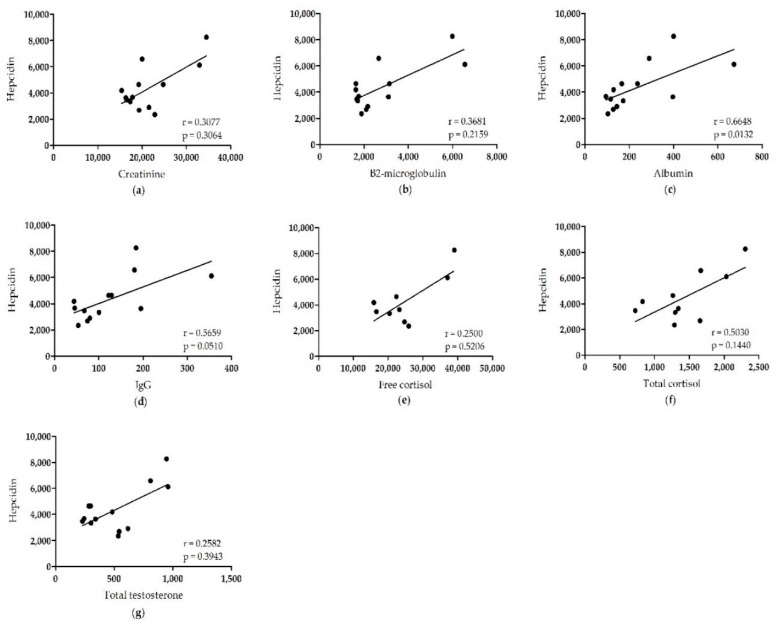
Correlations among the peritoneal clearance of hepcidin with all analytes in patients with end-stage renal disease undergoing a peritoneal equilibration test. The peritoneal clearance of hepcidin was correlated to the peritoneal clearance of (**a**) creatinine, (**b**) β2-microglobulin, (**c**) albumin, (**d**) IgG, (**e**) free cortisol, (**f**) total cortisol, and (**g**) total testosterone. The strength of the correlation was measured using Spearman’s correlation coefficient (r).

Last, we correlated the PC of all analytes with the PC of urea (Figure 2) to ensure that the variation in PC measurement of each analyte is solely caused by patient-specific transport characteristics. We have chosen urea for this since its PC measurements showed the lowest variation. We found a strong correlation between urea and creatinine (Spearman r = 0.9604, *p* < 0.0001), β2-microglobulin (Spearman r = 0.7890, *p* = 0.0008), free cortisol (Spearman r = 0.9333, *p* = 0.0002), total cortisol (Spearman r = 0.7697, *p* = 0.0092), and total testosterone (Spearman r = 0.6593, *p* = 0.0142). We observed a weaker correlation between urea and IgG (Spearman r = 0.5516, *p* = 0.0408) and urea and albumin (Spearman r = 0.5077, *p* = 0.0638). However, we did not find any correlation between urea and hepcidin (Spearman r = 0.2198, *p* = 0.4706). Further analysis showed that hepcidin does not correlate with any of the other analytes (Figure 3), except albumin (Spearman r = 0.6648, *p* = 0.0132). These results confirm a large inter-individual variation in hepcidin clearance, independent of peritoneal transport characteristics. Although this might influence the accuracy of the determined binding percentage, we conclude that a substantial proportion of hepcidin is freely circulating and exclude that circulating hepcidin is predominantly bound to plasma proteins.

## 3. Discussion

We applied the principle of PD to study the plasma protein binding of hepcidin in a functional human setting for the first time. To this end, we measured hepcidin along with known freely circulating and predominantly protein-bound solutes in blood and dialysate of patients with ESRD treated with PD undergoing PET. Our findings exclude that circulating hepcidin is predominantly bound to plasma proteins; however, the lack of correlation between hepcidin clearance and the clearance of the other analytes question the suitability of this model to determine an accurate binding percentage for this hormone.

Our finding that hepcidin is not predominantly bound is in line with other literature, in which a merely freely circulating hepcidin was observed using gel chromatography on human serum samples, followed by Liquid Chromatography-Mass Spectrometry (LC-MS/MS) [12]. In fact, other peptide hormones such as human growth hormone [27], insulin [28], and cardiac natriuretic hormones [29] circulate freely, since peptide hormones are often water-soluble and can, therefore, easily be transported via the cardiovascular system without requiring carrier proteins [30,31]. In addition, hepcidin is rapidly excreted by the kidneys, leading to a short half-life of only several hours [32,33,34]. Moreover, circulating hepcidin concentrations can change rapidly with subsequent effects on the circulating iron levels within 1–2 h [35,36]. Theoretically, this potential of a fast production [33,35,37] and quick turnover of hepcidin would preclude the need for protein binding [30].

Our observations differ from previous findings describing high hepcidin binding [10,11]. We believe this reported high binding percentage of ≈89% might be attributed to the excess of hepcidin (i.e., 100 times higher than physiological conditions) that was used in the experimental set-up, in combination with physiological α2M concentrations. This might have caused nonspecific binding of hepcidin, which the molecule is prone to due to its amphipathic character [38].

To the best of our knowledge, there are currently no established in vivo models to study protein binding and free drug or biomarker concentrations, which makes our explorative approach to study the functional protein binding of hormones unique. PET is regularly used as diagnostic test to characterize the functionality and transport properties of the peritoneal membrane in PD patients. Therefore, the strength of using PET is that dialysate sampling for research purposes provides human data with no additional burden for patients and it is a relatively simple, quick, and affordable method. Albeit we circumvent the disadvantages of in vitro techniques used to assess free hormone concentrations in diagnostic medicine, several limitations of our method were discovered during the study.

First, hepcidin did not correlate with urea or any of the other analytes, except albumin, suggesting that other variables besides patient-specific peritoneal transport characteristics may have affected hepcidin PC quantification. This raises the question if we can infer an accurate binding percentage for hepcidin, despite the fact that the validity of our model is confirmed by total cortisol and testosterone measurements and deduction of their binding percentages.

One of the possible explanations for the lack of correlation is the circadian rhythm of hepcidin, with plasma levels increasing during the day [39,40,41,42,43]. Due to practical concerns, blood and dialysate samples were not collected at the same time point, which could influence the determined hepcidin clearance differently for each patient. Nonetheless, in our previous study [43], the serum hepcidin median increased only by 2.5% between 11:00 and 13:30 amongst 24 participants. Therefore, the influence of hepcidins circadian rhythm seems unlikely since we used a time interval of only 1 h between blood and dialysate collection and PET start times were between 8:30 and noon. In addition, we did find a strong correlation between urea and both free and total cortisol, a hormone which also follows a circadian rhythm [44], suggesting the influence of a circadian rhythm is negligible.

Second, our study population suffers from ESRD and may therefore differ from healthy subjects, although there is no data to support the idea that peritoneal transport is affected by kidney function. It is described, however, that renal failure leads to the accumulation of uremic toxins in blood and tissues. These toxins can compete with other substrates for plasma protein binding to, for example, albumin [45]. As a result, the free fraction of a substrate of albumin will be increased. The strong correlation between hepcidin and albumin could imply binding of hepcidin to albumin and, therefore, the presence of uremic toxins might explain the observed inter-individual variability between hepcidin measurements. This might have led to an overestimation of the free fraction of hepcidin. However, testosterone and cortisol are also partially bound to albumin and we see no effect on their measurements. Therefore, interference of uremic toxins appears less likely. Nonetheless, we suggest our data should be validated and confirmed in healthy individuals or patients with a normal glomerular filtration rate.

Third, we cannot exclude our results are affected by production of hepcidin by mesothelial cells of the peritoneal membrane [46]. However, we feel that this is rather unlikely due to the high volume (2000 mL) new dialysate infused at t = 0 and the short dialysate time of 60 min.

Fourth, it is known that macromolecules may be removed from the peritoneal cavity by lymphatic absorption, which is independent of molecular size [47]. We observed a weak correlation between the small molecule urea and both macromolecules albumin and IgG. This might indicate that transport of these molecules is not solely dependent on permeability of the peritoneum, but also on lymphatic absorption, which would influence the established curve and could possibly introduce the observed inter-individual variation.

Last, clearance measurements of both hepcidin and albumin might be affected by their adherence to the dialysis bag, since both molecules possess nonspecific binding characteristics [38,48]. Due to their amphipathic character and suggested subsequent adherence to laboratory plastics, an extra factor of inter-individual variation is introduced, independent of patient specific peritoneal transport characteristics. This could explain the lack of correlation with other analytes. Regarding the assessment of hepcidins binding percentage, this would imply that the hepcidin concentration in dialysate, and therefore its clearance, might be underestimated and the free fraction would actually be higher.

Knowledge on the binding percentage of hepcidin is of great importance for correct quantification of the bioactive hormone, which in turn is key for both interpretation and standardization of hepcidin measurements in diagnostics of iron related disorders [1]. Characterization of hepcidin as measurand will add a key aspect to its metrological traceability chain that has recently been established, which is needed to obtain global uniform reference intervals and clinical decision limits for diagnostics [13]. Furthermore, correct analysis and interpretation of hepcidin measurement is essential in target assessment of clinical trials with hepcidin agonists and antagonists. These therapeutics are in development for treatment of several iron metabolism disorders such as hereditary hemochromatosis or anemia of inflammation [1,14]. A strong correlation has been observed between hepcidin-25 results of a wide variety of assays [13,49,50]. However, possible protein-binding could be an issue in correct hepcidin quantification when measuring target engagement of hepcidin antagonists that directly bind hepcidin in the circulation. These compounds display a high affinity for hepcidin and therefore specifically for the assessment of hepcidin antagonist efficiency, the remaining free, non-antagonist bound hepcidin should be quantified [51] rather than total hepcidin [52,53,54].

We can conclude that hepcidin is not predominantly bound and suggest that a substantial proportion is freely circulating, enabling direct and rapid bioactivity of the hormone. This is an important step towards unraveling human hepcidin plasma protein binding, although refinement of the model by inclusion of more patients and using more analytes with different MWs could help improve accuracy of our findings. Thereby, analytes with a high MW should preferably be excluded to circumvent influence of lymphatic absorption. Moreover, to definitively unravel hepcidin plasma protein binding, future studies must include studies on hepcidin production by mesothelial layer of the peritoneum and the prevention of nonspecific adherence of hepcidin in pre-analysis, which currently hampers (standard) dialysis techniques in diagnostic medicine.

## 4. Materials and Methods

### 4.1. Sample Collection

PET was performed according to the protocol of Isala hospital, Zwolle, The Netherlands. After an 10–12 h overnight dwell, a 2-Liter bag of 3.86% glucose solution (Baxter Healthcare Ltd., Newbury, UK) was instilled and allowed to dwell for 4 h [18]. Dialysate sampling was performed at 1 h of dwell time (t = 1 h), since peritoneal transport of solutes with a low MW (<200 Da) will decrease over a longer period of time due to saturation of the dialysate [22]. For sampling, all dialysate fluid was collected in a plastic (PVC) bag and mixed ex vivo before a sample was taken, to assure dialysate samples were representative for the fluid in the peritoneal cavity. Thereafter, the remaining dialysate was returned into the peritoneal cavity. Dialysate from 14 consecutive patients with ESRD who underwent PD was collected in tubes both with and without bovine serum albumin (1 g/50 mL, Sigma-Aldrich, Saint Louis, MO, USA), as for measurements of hepcidin and IgG, addition of albumin prevents adhesion to the tubes and subsequent loss. Blood samples were drawn prior to dialysis (t = 0 h) in both heparin plasma and serum tubes. All were centrifuged at 2000 g for 10 min. Blood and dialysate samples were aliquoted and immediately stored at −80 °C until measurement.

### 4.2. Laboratory Measurements

We measured total hepcidin concentrations (i.e., the sum of bound and unbound full length hepcidin-25), as well as freely circulating solutes urea, creatinine, β2-microglobulin, albumin, IgG, total testosterone (free and protein-bound), total cortisol (free and protein-bound), and free cortisol in blood and dialysate.

Analytical methods are described in Appendix A. All measurements were performed in freshly thawed aliquots within 8 months after collection.

### 4.3. Ethics

The study was approved by the Ethics Committee and the Board of Directors of Isala hospital, Zwolle, The Netherlands, and has been in accordance with the Helsinki Declaration. All patients signed informed consent prior to participation and all samples were blinded.

### 4.4. Calculations and Statistical Analysis

Measurement results for solutes below the lower limit of detection (LLOD) were excluded. We calculated PC (µL/min) for each analyte using the dialysate sample taken at t = 1 h and blood sample taken at t = 0 h of each analyte, using the patient-specific dialysate volume and a dialysis time of 60 min as:

Peritoneal clearance (µL/min) = [Dialysate]/[Blood] × (Volume dialysate)/(Duration of dialysis).(2)

Logarithmic transformation was used for PC data before statistical analysis, as the data was not normally distributed. In addition, we used a cube root scaling for MW, since transport rates are related to size and thus radius (r), and considering volume as a three-dimensional characteristic of the radius (r^3^). The curve describing the relation between MW and PC of all analytes was estimated with a linear mixed model using a random slope and random intercept. The expected PC of each analyte could be calculated using its MW and the equation of the curve. We calculated the binding percentage as:

Binding percentage (%) = (1 − (Measured PC)/(Expected PC)) × 100%.(3)

In this equation, the measured PC divided by the expected PC represents the free hormone fraction. The strength of a linear association between the clearances of two analytes was measured using Spearman’s correlation coefficient.

All statistical analyses were performed with SAS software, version 9.4 (SAS Institute, Inc. Cary, NC, USA) and GraphPad Prism 5.03 (GraphPad Software Inc., La Jolla, CA, USA).

## Figures and Tables

**Table 1:** Patient characteristics.

ID	Age (yrs)	Gender (M/F)	Underlying Disease	Use of Prednisone ^1^	Creatinine Clearance (L/week/1.73 m^2^) Total (residual)	Start Time PET (a.m.)
1	73	F	Primary amyloidosis	No	61.1 (32.3)	10:00
2	82	M	Renal vascular disease due to hypertension	No	56.4 (25.7)	09:30
3	55	F	Lupus erythematosus	1 × 7.5 mg	99.3 (73.7)	10:00
4	74	M	Renal vascular disease due to hypertension	No	68.2 (34.5)	08:30
5	72	M	IgA nephropathy	No	118.2 (69.6)	10:00
6	64	M	Renal vascular disease type unspecified	1 × 7.5 mg	70.5 (37.1)	12:00
7	66	M	Diabetes mellitus type 2	No	100.8 (63.9)	10:15
8	53	M	Chronic renal failure etiology unknown/uncertain	No	71.7 (43.2)	12:00
9	76	M	Renal vascular disease due to hypertension	No	114.0 (75.7)	12:00
10	72	M	Renal vascular disease type unspecified	No	128.6 (100.0)	10:00
11	55	F	IgA nephropathy	2 × 5 mg	122.6 (77.8)	11:30
12	61	F	Renal vascular disease type unspecified	1 × 5 mg	129.5 (96.7)	11:00
13	64	F	Diabetes mellitus type 2	No	101.8 (35.9)	09:30
14	77	M	Renal vascular disease due to hypertension	No	118.1 (73.1)	09:00

^1^ Since use of prednisone was found to decrease the serum cortisol concentrations and interferes with measurement of both free and total cortisol, these measurements were not performed in patients using prednisone.

**Table 2:** Molecular weight, measured plasma, and dialysate concentrations (mean ± sd) and subsequent calculated peritoneal clearances (mean ± sd) for all solutes.

Solute	MW (Da)	[Plasma] t = 0 h Mean (sd)	[Dialysate] ^1^ t = 1 h Mean (sd)	Peritoneal Clearance (µL/min) t = 0–1 h Mean (sd)	N
Urea (mmol/L)	60.0	21.5 (6.6)	15.0 (6.1)	26,959.1 (6211.8)	14
Creatinine (µmol/L)	113.1	542.0 (172.6)	296.9 (119.9)	21,499.6 (5861.6)	14
Testosterone, total (nmol/L) ^2^	288.0	8.6 (7.3)	0.1 (0.1)	507.7 (260.5)	13 ^3^
Cortisol, total (nmol/L) ^4^	362.5	229.1 (134.4)	8.4 (5.8)	1439.0 (490.8)	10 ^5^
Cortisol, free (nmol/L)	362.5	12.7 (7.7)	8.4 (5.8)	25,030.8 (8123.9)	9 ^5,6^
Hepcidin (nmol/L)	2789.4	10.6 (9.1)	1.2 (1.1)	4337.5 (1713.1)	13 ^7^
β2-microglobulin (mg/L)	13,713.0	18.7 (5.0)	1.4 (0.9)	2804.0 (1587.8)	14
Albumin (g/L)	66437.0	32.1 (4.9)	0.2 (0.1)	242.3 (164.2)	14
IgG (mg/L)	150,000.0	7496.0 (1730.3)	23.1 (12.7)	129.3 (84.7)	14

^1^ Average dialysate volumes were 2364 mL (range 1500-2250 mL). ^2^ Approximately 2% of the total plasma testosterone is circulating free in both men and women [9,26]; in men 44–65% of testosterone is bound to sex hormone binding globulin and 33–54% bound to albumin; in women 66–78% of testosterone is bound to sex hormone binding globulin (MW 90 kDa), and 20–32% to albumin (MW 66,437 Da) [9]. ^3^ One patient was excluded since dialysate concentrations of testosterone were below the LLOD. ^4^ Approximately 5% of the total plasma cortisol is circulating free, since 80–90% of cortisol is bound to corticosteroid binding globulin (MW 52 kDa) and 10–15% to albumin (MW 66,437 Da) [6]. ^5^ Patient 3, 6, 11, and 12 used prednisone, which interferes with cortisol measurement. The results of these patients were excluded. ^6^ For one patient material was insufficient to measure free cortisol. ^7^ One patient was excluded since both plasma and dialysate concentrations of hepcidin were below the LLOD.

## Appendix A

### Laboratory Chemistry Measurements

Measurements of urea, creatinine, and IgG were performed on a Cobas C8000 random access analyzer (Roche Diagnostics, Basel, Switzerland). Albumin was measured by nephelometry on a BNII system (Siemens, München, Germany). β2-microglobulin was measured by a direct sandwich-ELISA, using two polyclonal rabbit anti-human β2-microglobulin antibodies, of which one was HRP labeled. The pH of all peritoneal dialysate samples was 7.5 allowing accurate measurements of β2-microglobulin.

### Measurement of Hepcidin in Serum and Peritoneal Dialysate

Measurements of hepcidin were performed by a combination of weak cation exchange chromatography and time-of-flight mass spectrometry (WCX-TOF MS) using a stable hepcidin-25^+40^ isotope as internal standard for quantification [55]. This MS-based assay determines total hepcidin (i.e., the sum of bound and unbound fraction). Peptide spectra were generated on a Microflex LT matrix-enhanced laser desorption/ionisation TOF MS platform (Bruker Daltonics, Hamburg, Germany). Hepcidin concentrations were expressed as nmol/L. The lower limit of quantification of this method was 0.5 nM.

### Measurement of Total Cortisol and Total Testosterone in Serum

Cortisol and testosterone were analyzed by Liquid Chromatography-Mass Spectrometry (LC-MS/MS) after protein precipitation and solid-phase extraction. Internal standard [^2^H_4_]-cortisol and [^13^C_3_]-testosterone (Isoscience, Ambler, PA, USA) was added to 100 µL serum. Subsequently, 300 µL acetonitrile with 0.1% formic acid was added for protein precipitation and 300 µL H_2_O was added to 200 µL supernatant followed by solid phase extraction (Oasis HLB 1cc, Waters, Milford, MA, USA). The eluate (methanol:isopropanol 95:5) was dried under a stream of N_2_ gas, reconstituted in methanol:water (3:7) and injected (10 µL) into an Agilent Technologies 1290 Infinity Ultra High Performance Liquid Chromatography (UHPLC)-system (Agilent Technologies, Agilent, Santa Clara, CA, USA) equipped with a BEH C18 (1.7 μm 2.1 × 50 mm) analytical column (Waters Corp.) at 60 °C. Mobile phase A (methanol:water 20:80 + 2 mM NH_4_CH_3_COO + 0.1% formic acid) and B (methanol:water 98:2 + 2 mM NH_4_CH_3_COO + 0.1% formic acid) were run in a gradient (0.4 mL/min). Start gradient 70:30 A:B for 2.5 min; then to 40:60 A:B in 3.5 min; followed by a gradient in 0.5 min to 2:98 to remain such for 0.5 min and thereafter to 70:30 A:B in 0.5 min and remain such for 0.5 min. Retention time was 1.41 min and 4.37 min for cortisol and testosterone, respectively. Total run time was 8 min. A 9-point calibration curve was used (cortisol and testosterone, Steraloids). An Agilent 6490 tandem mass spectrometer (Agilent Technologies, Agilent, Santa Clara, CA, USA) was operated in the electrospray positive ion mode, with a capillary voltage 3.5 kV, fragmentor voltage 380 V, sheath gas temperature 350 °C, and gas temperature 150 °C with N_2_ collision gas. Two transitions (qualitative and quantitative) were monitored. Transitions (Q1 > Q3) were m/z 363.4 > 97.1 (34 V) and m/z 363.4 > 121.1 (25 V) for cortisol; m/z 367.4 > 97.1 (34 V) and m/z 367.4 > 121.1 (25 V) for [2H4]-cortisol. m/z 289.2 > 109.1 (30 kEV) and m/z 289.2 > 97.1 (30 kEV) for testosterone; m/z 292.3 > 112.1 (30 kEV) and m/z 292.3 > 100.1 (30 kEV) for 13C3-testosterone. Dwell time was 100 ms and 50 ms for cortisol and testosterone, respectively. The method was linear assessed by CLSI EP6 protocol [56]. For cortisol total CV is 3.6% at 300 nmol/L and 3.1% at 1080 nmol/L. For testosterone total CV is 6.0% at 0.9 nmol/L and 5.3% at 19 nmol/L.

### Measurement of Free Cortisol in Serum by Equilibrium Dialysis—Liquid Chromatography Tandem Mass Spectrometry (LC-MS/MS)

Serum was dialyzed in a multi-cell equilibrium dialyzer using Teflon dialyzing cells consisting of two identical parts between which a flat membrane is fitted (Dianorm-Geräte). The dialysis cells are incubated in a water bath with constant agitation by a rotating apparatus and temperature during dialysis was maintained at 37 ± 0.5 °C, by constant monitoring with an immersion thermostatic system (Tinytag TGP-4020 logger and PB5001-1M5 sonde; Gemini data loggers). The dialysis membranes were prepared from Dianorm (Diachema dialysis membranes 10.14) with a cut-off of 5 kDa. Equilibrium dialysis was performed for 5 h with 180 µL serum against 180 µL HEPES buffer; while rotating the dialysis apparatus at 20 rpm. The HEPES buffer used for equilibrium dialysis was composed to reflect the electrolyte composition and pH of serum and contained in addition to 12.570 g/L N-(2-hydroxyethyl)piperazine-N’-(2-ethanesulfonic acid (HEPES), 5.353 g/L NaCl, 0.200 g/L KCl, 0.224 g/L KH_2_PO_4_, 0.275 g/L MgSO_4_·7 H_2_O, 0.300 g/L urea, 0.275 g/L CaCl_2_·2 H_2_O, 0.900 g/L NaOH, and 0.520 g/L NaN_3_ (Merck, Darmstadt, Germany). The pH was adjusted with HCl to pH 7.40 (at 37 °C). The buffer was diluted by ultrapure water produced with a Milli Advantage A10 and 100 µL dialysate was subsequently used for total cortisol measurement by LC-MS/MS as described above.

### Measurement of Total Cortisol and Total Testosterone in Peritoneal Dialysate

Testosterone and cortisol in peritoneal dialysate were analyzed by LC-MS/MS after solid-phase extraction. Sample preparation consisted of internal standard addition [13C3]-testosterone (Isoscience, Ambler, PA, USA) and [^3^H_4_]-cortisol (Isoscience) to 1000 µL dialysate and solid phase extraction (SPE) using Oasis^®^ MCX 1cc cartridges (Waters Corp, Milford, MA, USA).

Columns were pre-equilibrated with 1 mL MeOH:IPA (95:5) and subsequently washed with 1 mL H_2_O. After application of the sample, columns were washed with 1 mL H_2_O:NH_4_OH (95:5) and 1 mL MeOH:H_2_O (20/80) + 2% HCOOH. The 300 µL eluate (MeOH) was dried under a stream of N_2_ gas, reconstituted in MeOH:H_2_O (30:70). A nine-point calibration series was used (using testosterone and cortisol both from Steraloids). Calibrators, quality controls, and samples (reconstituted in 100 µL) were injected (10 µL) into an Agilent Technologies 1290 Infinity VL UHPLC-system (Agilent Technologies, Santa Clara, USA), equipped with a BEH C18 (1.7 μm 2.1 X 50 mm) analytical column (Waters Corp., Milford, MA, USA) at 60 °C. Mobile phase A (MeOH/H_2_O (20:80) + 2 mM NH_4_CH_3_COO + 0.1% HCOOH) and B (MeOH/H_2_O (98:2) + 2 mM NH_4_CH_3_COO + 0.1% HCOOH) were run in a gradient (0.4 mL/min). The gradient program was as follows: Start gradient A:B (70:30) for 2.5 min; followed by a gradient in 2 min to A:B (60:40) and subsequent 2.5 min A:B (35:65). For column washing, a gradient 0.5 min A:B (98:2) was established to remain such for 0.5 min. The column was re-equilibrated to A:B (70:30) in 0.5 min and remained such for 0.5 min. Retention times were 1.5 min and 4.5 min for cortisol and testosterone, respectively. An Agilent 6490 tandem mass spectrometer (Agilent Technologies, Santa Clara, CA, USA) was operated in the electrospray positive ion mode. Operating conditions were as follows: Capillary voltage 3.5 kV, fragmentor voltage 380 V, sheath gas temperature 350 °C, and gas temperature 150 °C. The collision energy was optimized between 25 and 34 eV for all solutes and Nitrogen was used as collision gas. Two mass transitions were monitored for each analyte and the internal standards. The first transition was used for quantification, the second for confirmation. The transitions (Q1 > Q3) were m/z 289 > 97 and m/z 289 > 109 for testosterone; m/z 292 > 100 and m/z 292 > 112 for [13C3]-testosterone; m/z 363 > 121 and m/z 363 > 97 for cortisol; m/z 367 > 121 and m/z 367 > 97 for [3H4]-cortisol. Total run time was 9 min.

For both total and free cortisol PC calculations, we used total cortisol measurements in dialysate, since we assume that all cortisol present in dialysate after 1 h of dwell time is free considering the large MW of its main binding protein corticosteroid binding globulin (MW 52 kDa).
